# Frailty worsens long-term survival in patients with colorectal cancer: a systematic review and meta-analysis

**DOI:** 10.3389/fonc.2024.1326292

**Published:** 2024-02-09

**Authors:** Jiangxue Han, Qin Zhang, Jiarong Lan, Fang Yu, Jie Liu

**Affiliations:** ^1^ Oncology Department, Jiaxing Hospital of Traditional Chinese Medicine, Jiaxing, China; ^2^ School of Basic Medical Sciences, Zhejiang Chinese Medical University, Hangzhou, China; ^3^ Department of Medicine, Huzhou Traditional Chinese Medicine Hospital Affiliated to Zhejiang Chinese Medical University, Huzhou, China; ^4^ Department of Pathology, Jiaxing Hospital of Traditional Chinese Medicine, Jiaxing, China; ^5^ Institute of Integrated Chinese and Western Medicine, Shanghai University of Traditional Chinese Medicine, Shanghai, China

**Keywords:** carcinoma, geriatric, mortality, recurrence, frail

## Abstract

**Background:**

Colorectal cancer (CRC) is the 3^rd^ most common cancer in men and 2^nd^ most common malignancy in females across the globe leading to high mortality rates. Frailty is an age-related syndrome that has been associated with high morbidity and mortality. This systematic review aimed to examine if frailty can predict long-term (>1 year) outcomes of patients with CRC.

**Methods:**

This PROSPERO registered review examined the databases of PubMed, Embase, and Web of Science till 4^th^ September 2023 for cohort studies assessing the association between frailty and long-term outcomes of CRC.

**Results:**

15 studies with 45288 patients were included. 6573 patients (14.5%) were frail. Meta-analysis demonstrated that frailty was associated with statistically significant poor overall survival (OS) (HR: 2.11 95% CI: 1.44, 3.08 I^2^ = 94%) (14 studies), cancer-specific survival (CSS) (HR: 4.59 95% CI: 2.75, 7.67 I^2^ = 38%) (2 studies), and disease-free survival (DFS) (HR: 1.46 95% CI: 1.28, 1.66 I^2^ = 0%) (5 studies) after CRC. Subgroup analysis for OS based on study type, location, sample size, stage of cancer, percentage with frailty, treatment, adjustment for CRC stage and comorbidities, and follow-up did not change the results. These results were not altered in significance on sensitivity analysis.

**Conclusion:**

Our results show that frail CRC patients have poor OS and DFS as compared to non-frail patients. Variations in frailty measurement tools and high inter-study heterogeneity are major limitations of the review.

**Systematic review registration:**

https://www.crd.york.ac.uk/prospero/, PROSPERO, CRD42023450586

## Introduction

Colorectal cancer (CRC) is one of the most prevalent malignancies diagnosed worldwide. In men, it stands as the 3^rd^ most common cancer while in females it is the 2^nd^ most common malignancy across the globe (1). Some geographical variations have been noted in its incidence with a predominance in Asian populations but in recent times a large number of cases have also been noted in Western populations (2). Additionally, CRC has a predilection for the elderly with more than 60% of cases being detected in those aged ≥65 years and the median age of diagnosis is 67 years (3).

Elderly patients constitute a special cohort due to age-related reduction in host immunity and organ functions. The diminished physiologic reserve seen with increased age which causes a reduction in resiliency and adaptive capacity and increased vulnerability to stressors has been termed as frailty (4). It is characterized by muscle wasting, weight loss, and a decline in the functional capacity of the individual. Frailty, which can be classified as an age-related syndrome, has been associated with high morbidity and mortality (5). Additionally, such patients have a higher risk of surgical complications (4) and chemotherapy-related toxicities (6). Multiple studies have shown that frailty is seen in about 5-71% of elderly patients (7, 8). Frailty has also been associated with malnutrition and chronic inflammation. Together with the increased risk of treatment-related complications, frailty may also be associated with poor long-term survival and increased risk of recurrence after cancer (7, 8). Indeed, frailty can be a good prognostic indicator performing better than other indices like the American Society of Anesthesiology score or the Charlson Comorbidity Index (9, 10).

The association between frailty and outcomes of CRC has been examined by many studies but most have focused on short-term outcomes (4). There is a paucity of data on the impact of frailty on long-term outcomes of CRC. Therefore, we performed the current systematic review and meta-analysis to compile data from published studies and provide the best possible evidence on the prognostic role of frailty in cases of CRC.

## Methods

### Search strategy and inclusion criteria

As per guidelines, the reviewers drafted a review protocol and uploaded it on the international register PROSPERO (Available on https://www.crd.york.ac.uk/prospero/ with registration number CRD42023450586). The study was performed based on the criteria of the Preferred Reporting Items for Statistic Reviews and Meta-Analyses statement (11). Databases of PubMed, Embase, and Web of Science were searched online for English-language articles. The search was completed on 4^th^ September 2023. Conference proceedings and unpublished or non-peer-reviewed data were not considered during the search.

All eligible studies had to meet the following criteria: 1. Cohort studies conducted on adult CRC patients. 2. Reporting association between frailty and overall survival (OS) or disease-free survival (DFS). 3. The examined association was reported as an effect size with 95% confidence intervals (CI) or sufficient data was provided to calculate the same. 4. Follow-up was at least one year. All criteria for evaluation of frailty were acceptable.

Studies not reporting separate data for CRC, reporting only short-term outcomes, and duplicate studies were excluded.

The search strategy was formulated using the keywords: “frail”, “frailty”, “geriatric assessment”, “colorectal cancer”, “colon cancer”, “rectal cancer”, “colorectal carcinoma”, “colon carcinoma”, and “rectal carcinoma”. Different combinations were used using AND and OR.

Two reviewers were involved in the search process which first began with title and abstract screening. Studies were excluded if the title or the abstract did not conform with the aims of this review. Full text was then obtained for all identified acceptable studies, or when the relevance of an article could not be determined. Disagreements were settled by consensus. The same process was performed for the full-text review. The bibliography of included studies was cross-referenced to discover further eligible studies.

### Data extraction

Two reviewers performed the data extraction and assimilated information related to the year of the study, the author’s first name, country, study type, sample size, mean/median age of the population, gender details, CRC stage, frailty scale, number of frail patients, treatment protocols used, factors adjusted in the outcome analysis, and follow-up.

According to the guidelines of the Newcastle-Ottawa Scale (NOS) (12), the included studies were judged for bias by two independent reviewers in the domains of selection of cohort, comparability, and outcome assessment. The three components were given points for questions included in the NOS. The total points available are; selection: 4; comparability: 2; and outcome assessment: 3.

### Statistical analysis

Outcomes assessed by quantitative analysis included OS, DFS, and cancer-specific survival (CSS). The effect size for the association between frailty and survival was combined to generate a hazard ratio (HR) with 95% CI. If the effect size was not reported in numerical values in any article, the same was calculated from Kaplan-Meier survival plots (13). Meta-analysis was conducted in a random-effects model using the software “Review Manager” (RevMan, version 5.3). Outliners were assessed using a sensitivity analysis involving the removal of one study at a time. The chi-square-based Q statistics and I^2^ statistics were used for inter-study heterogeneity. A p-value of <0.10 for Q statistic and I^2^ >50% meant substantial heterogeneity. A funnel plot was drawn to examine publication bias. Subgroup analyses were conducted for OS based on study type, location, sample size, stage of cancer, frailty scale, percentage with frailty, treatment, adjustment for CRC stage and comorbidities, and follow-up. Additional subgroup analyses could not be conducted for CSS and DFS due to the paucity of data.

## Results

**Figure 1:**
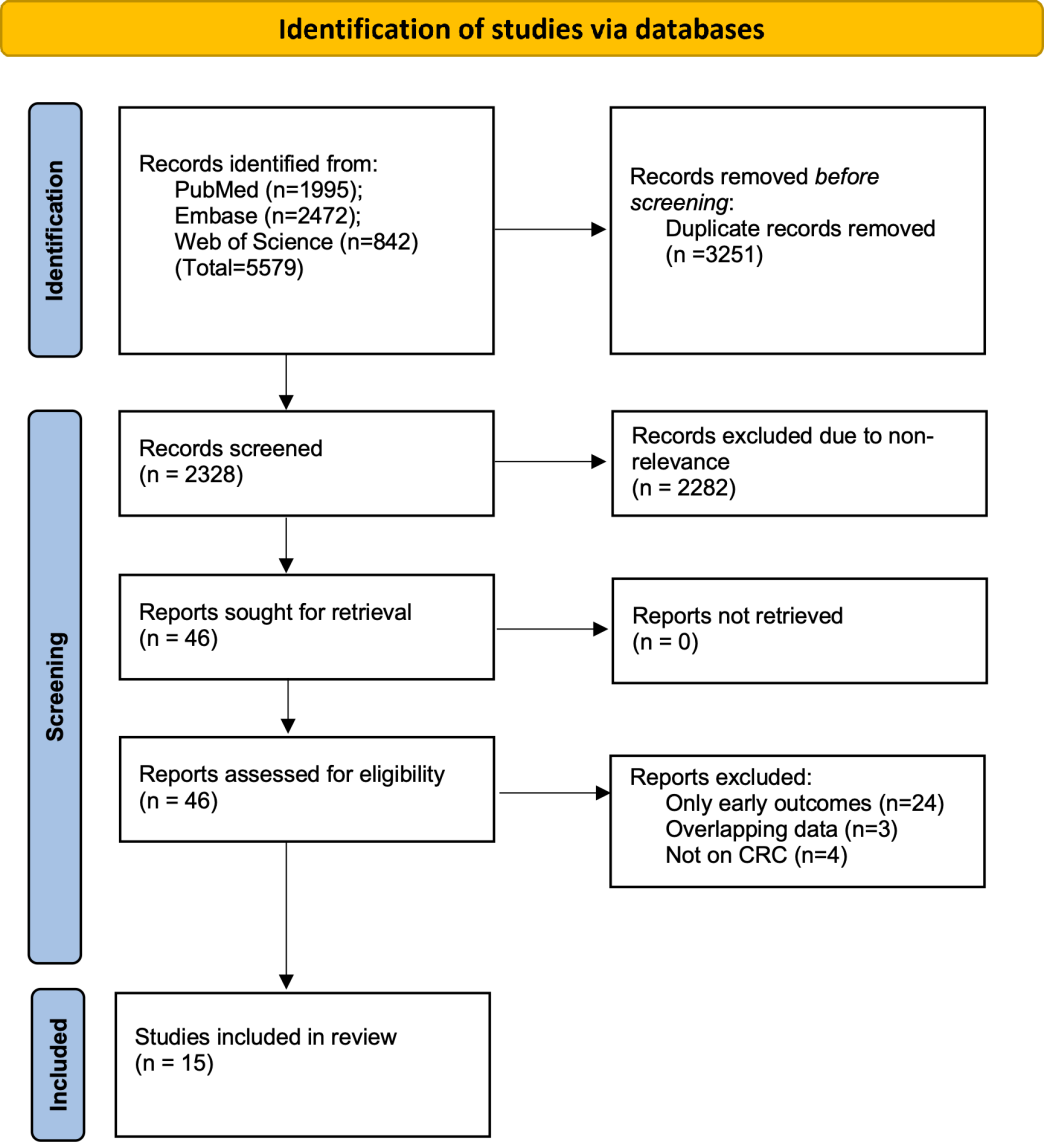
Flowchart of the study depicting search results at every stage.

The two reviewers found 5579 articles from the databases. After electronic deduplication, 2328 were screened and 46 articles were identified by the reviewers for further analysis. The inter-reviewer rating for the selection of studies was high (kappa= 0.9). Finally, based on the inclusion criteria, 15 studies were included in the review (14–28) (**Figure 1**).

Characteristics of the included studies are presented in **Table 1**. Seven were retrospective cohort studies while the rest were prospective. All of them were published in the past decade (2013–2023). The studies originated from the USA, Scandinavia, the Netherlands, Italy, Spain, Germany, Japan, and Australia. The combined sample size of the 15 studies was 45,288 of which 6573 patients (14.5%) were classified as frail. Individually the percentage of frail patients in the studies ranged from 8.3 to 52.2%. The frailty assessment scale varied amongst the included studies. Two studies did not report the treatment protocols used. In five studies, it was surgery and chemotherapy while in the remaining studies, it was surgery only. Except for one study, all studies reported an adjusted association between frailty and OS/DFS after CRC. However, the confounders adjusted in the analysis were variable. Follow-up ranged from 1-5 years. Seven studies received an NOS score of 8, seven studies got a score of 7, and one study had a score of 6.

**Table 1:** Extracted data from included studies.

Study	Year	Location	Type	Sample size	Mean/Median Age (years)	Male (%)	Cancer stage	Frailty scale	% with frailty	Treatment	Adjusted factors	Follow-up (years)	NOS score
Neuman et al (14)	2013	USA	R	31574	85.2	36.6	I-III	The Johns Hopkins University Risk Adjustment Model and ICD-codes	8.3	Surgery	Age, sex, race, health factors, mobility, comorbidities, and receipt of surgery	1	8
Aaldriks et al (15)	2013	The Netherlands	P	143	75	59	II-IV	Groningen Frailty Index	23.7	Surgery, Chemotherapy	Age, sex, number of comorbidities, hemoglobin, creatinine and lactate dehydrogenase	1.3	8
Ommundsen et al (21)	2014	Norway	P	178	80	43	I-IV	CGA based criteria	42.7	Surgery	Age, sex, and tumor stage	4.8	8
Jorgensen et al (23)	2015	Australia	R	1483	NR	49.1	I-IV	ICD codes	46.4	Surgery, Chemotherapy	Age, hospital admission factors, and number of comorbidities	1	7
Ugolini et al (22)	2015	Italy	P	46	80.5	52.2	I-III	Groningen Frailty Index	52.2	Surgery	None	4.6	6
Bensken et al (16)	2020	USA	R	5462	NR	48.7	I-IV	Claims Frailty index	19.4	NR	Age, sex, cancer stage	1.5	7
Feliciano et al (26)	2020	USA	P	691	NR	0	I-IV	Fried Frailty scale	15.9	NR	Age, race/ethnicity, body mass index, smoking, educational attainment, CCI, any family history of cancer, and cancer stage	5.8	8
Mima et al (25)	2020	Japan	R	729	NR	53	I-III	Clinical Frailty scale	34.7	Surgery, Chemotherapy	Age, physical status, carcinoembryonic antigen, carbohydrate antigen 199, tumor stage, and adjuvant chemotherapy	3.5	7
Viles et al (24)	2020	The Netherlands	P	466	75	59.1	I-IV	Geriatric 8 questionnaire	41.8	Surgery	Age, sex, and physical status	2.1	8
Artiles et al (28)	2021	Spain	P	149	75	64.4	I-IV	Clinical Frailty scale	39.6	Surgery	Age, CCI, and stage	5	7
Tokuda et al (27)	2021	Japan	R	87	68	63.2	IV	Clinical Frailty scale	33.3	Surgery	Age, sex, modified Glasgow Prognostic Score, tumor size, carcinoembryonic antigen, carbohydrate antigen 199	3.9	8
Chen et al (17)	2022	Germany	P	3410	70	60.6	I-IV	Mitnitski and Rockwood method	35.6	Surgery	Age, sex, cancer stage and cancer location	5	8
Abdelfatah et al (19)	2023	USA	R	411	75.1	49.4	I-IV	revised Risk Analysis Index	29.9	Surgery	NR	4	7
Liposits et al (20)	2023	Scandinavia	P	160	78	51	IV	Geriatric 8 questionnaire	28	Surgery, Chemotherapy	Age, sex, body mass index, and treatment allocation	2	7
Ogata et al (18)	2023	Japan	R	299	NR	57.6	I-III	five-factor modified frailty index	14.7	Surgery, Chemotherapy	NR	5	7

CCI, Charlson Comorbidity Index; R, retrospective; P, prospective; ICD, International Classification of Diseases; CGA, comprehensive geriatric assessment; NR, not reported.

**Figure 2:**
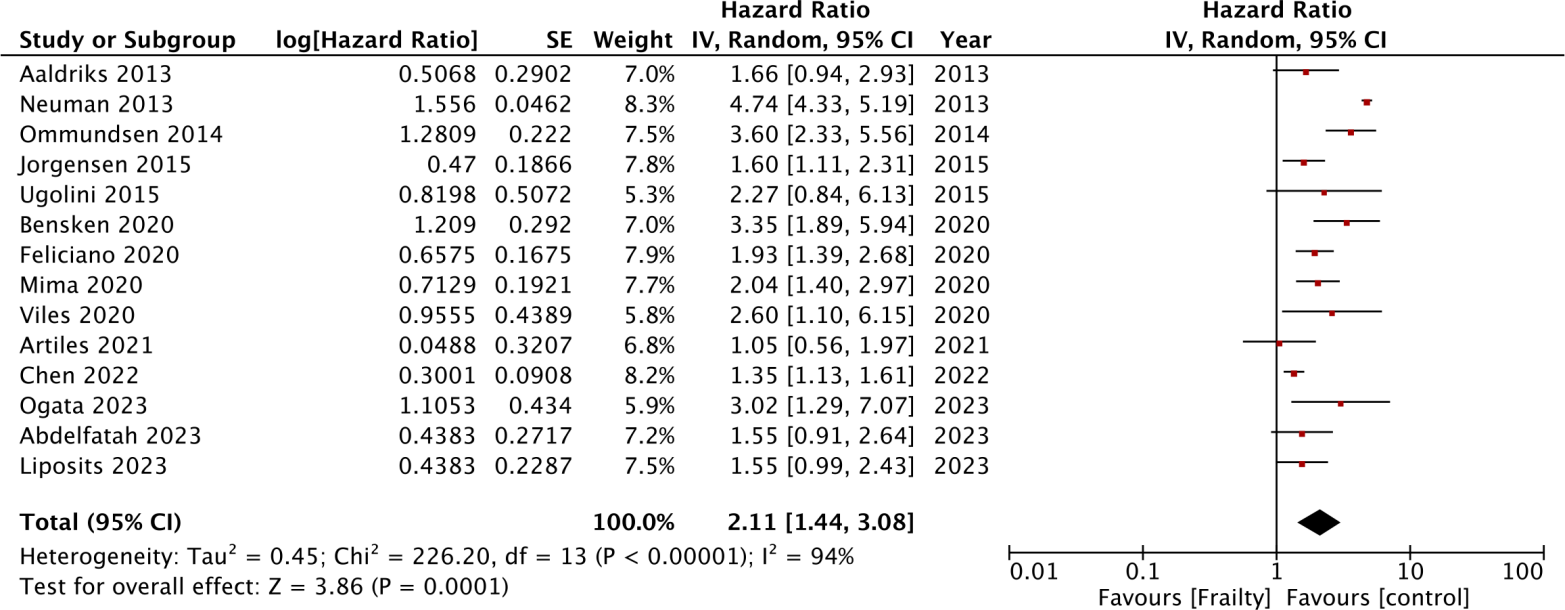
Meta-analysis comparing OS after CRC between frail and non-frail patients.

Meta-analysis of 14 studies demonstrated that frailty was associated with statistically significant poor OS in CRC (HR: 2.11 95% CI: 1.44, 3.08 I^2^ = 94%) (**Figure 2**). There was no major asymmetry on the funnel plot (**Supplementary Figure 1**). The reviewers also examined changes in the pooled effect size by sequentially withdrawing individual studies from the meta-analysis only to find no change in the significance of the outcome.

Data from the subgroup analyses conducted for OS are shown in **Table 2**. The results were statistically significant for poor OS on subgroup analysis based on study type (retrospective and prospective), study location (USA, Europe, and others), sample size (>1000 and <1000), stage (including or excluding stage IV), percentage of frailty in the population (>35% and <35%), treatment (surgery and surgery + chemotherapy), adjusted for cancer stage (yes and no), adjusted for comorbidities (yes and no), and follow-up (>3 years and <3 years). The results turned non-significant only on subgroup analysis based on frailty scales.

**Table 2:** Subgroup analysis for the outcome OS using different variables.

Variable	Groups	Studies	HR [95% CI]	I^2^
Study type	Prospective Retrospective	8 6	1.81 [1.37, 2.40] 2.50 [1.48, 4.23]	67 92
Location	USA Europe Others	4 7 3	2.67 [1.44, 4.95] 1.80 [1.28, 2.53] 1.89 [1.45, 2.45]	93 70 6
Sample size	>1000 <1000	4 10	2.41 [1.07, 5.42] 1.97 [1.58, 2.45]	98 40
Stage	Including stage IV Without stage IV	10 4	1.83 [1.45, 2.32] 2.98 [1.66, 5.34]	67 86
Frailty scale	Groningen Frailty Index Clinical Frailty scale ICD codes Geriatric 8 questionnaire	2 2 2 2	1.79 [1.09, 2.94] 1.53 [0.81, 2.92] 2.80 [0.97, 8.10] 1.75 [1.14, 2.70]	0 69 97 8
% with frailty	>35 <35	6 8	1.84 [1.26, 2.68] 2.30 [1.47, 3.59]	75 92
Treatment	Surgery Surgery plus chemotherapy	7 5	2.18 [1.17, 4.06] 1.78 [1.45, 2.18]	97 0
Adjusted for cancer stage	Yes No	6 8	2.00 [1.41, 2.85] 1.97 [1.14, 3.39]	81 93
Adjusted for comorbidities	Yes No	6 8	2.27 [1.33, 3.86] 1.96 [1.39, 2.76]	94 75
Follow-up	>3 years <3 years	8 6	1.89 [1.42, 2.52] 2.37 [1.35, 4.16]	70 92

CI, confidence intervals; ICD, International Classification of Diseases, I^2^, inter-study heterogeneity; HR, hazard ratio.

**Figure 3:**
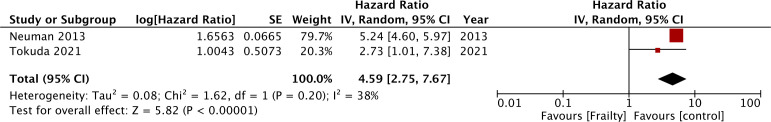
Meta-analysis comparing CSS after CRC between frail and non-frail patients.

**Figure 4:**
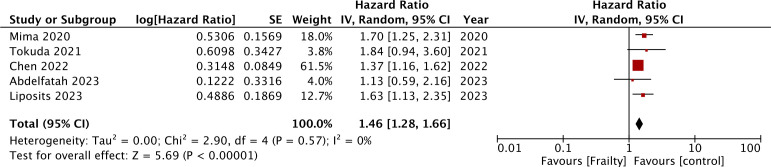
Meta-analysis comparing DFS after CRC between frail and non-frail patients.

Just two studies reported CSS and meta-analysis showed significantly poor CSS in CRC in the presence of frailty (HR: 4.59 95% CI: 2.75, 7.67 I^2^ = 38%) (**Figure 3**). Five studies reported data on DFS. Pooled analysis showed frailty was associated with statistically significant poor DFS in CRC (HR: 1.46 95% CI: 1.28, 1.66 I^2^ = 0%) (**Figure 4**). These results were not altered in significance on sensitivity analysis.

## Discussion

In recent times, there have been increased efforts to build and validate accurate prediction models for the prognosis of CRC (29). The Tumour Node Metastasis (TNM) staging classification system has been the primary prognostic tool in the hands of clinicians, however, many variations have been noted in OS and DFS even within stage groupings (30). The recent 7^th^ edition of the UICC/AJCC anatomic stage proposed anatomically-based subgroupings in stage II and III CRC to consider the differences in prognosis within these sub-groups (31). Nevertheless, there are still limitations and the prognosis of CRC can be further improved by including clinical, disease, and patient characteristics (32). In the current era of personalized medicine, patients’ functional status and frailty could be important predictors of outcomes and several studies have already shown that frailty is a predictor of short-term outcomes in CRC. Cai et al (4) in a systematic review of 18 studies have demonstrated that frailty measured by different scales did increase the risk of early mortality, serious complications, postoperative blood transfusions, and delirium in CRC irrespective of the treatment provided. While the role of frailty in predicting short-term outcomes has been thoroughly investigated, its impact on long-term outcomes has received limited attention.

Previously, Chen et al (33) have pooled data literature to demonstrate that frailty was associated with worse OS [relative risk (RR) 2.21 95% CI: 1.43, 3.41] and DFS (RR: 1.72, 95% CI 1.30-2.28) in CRC. However, their review could include only 10 studies and only two studies were available for the analysis of DFS. We conducted an updated literature search to include five more studies to provide the most recent evidence on this topic. The current study combined data from 15 studies which included mostly elderly patients with all stages of CRC and were treated with a mix of surgery and chemotherapy. Using different frailty scales, the studies noted that that about 14.5% patients were frail. Individually, the forest plot showed that frailty was associated with poor OS and DFS in all studies. On combined analysis, the meta-analysis confirms with fact that frailty is associated with statistically significant worse OS in patients with CRC. The HR demonstrated that frail patients had a two-fold increased risk of long-term mortality and the results did not change during sensitivity analysis thereby increasing the credibility of the outcomes. Pooled analysis of five studies also showed a statistically significant worse DFS in frail vs. non-frail patients with CRC indicating the fact that frail patients are at increased risk of both mortality and recurrence. Similar results have been reported for other cancers as well. Komici et al. (8) have shown that frailty leads to three times higher risk of mortality in lung cancer patients. A recent study by Tsai et al (34) has shown that frailty increases mortality by 1.5 and 2.9 times in metastatic and non-metastatic breast cancer. Another large study from Australia and New Zealand shows that frailty increases the risk of death by 52% in patients with metastatic cancers (35).

The high inter-study heterogeneity noted in the meta-analysis is a major cause of concern. However, this was partially expected as the inclusion criteria of the review were broad and there was no restriction placed on the cancer stage, treatment, and measurement of frailty. Also, there was variation in the quality of included studies with the NOS varying from 6 to 8. Some studies had longer follow-up periods and adjusted for more confounders which impacted the NOS score. The included studies had much variation in all these factors which was further investigated by a detailed subgroup analysis. It was noted that frailty consistently predicted poor OS irrespective of the study type, location, sample size, stage of cancer, number of patients with frailty, the treatment provided, and the follow-up duration. Secondly, the analysis on OS included mostly adjusted data from the included studies and only one study failed to adjust for confounders. The exclusion of this study on sensitivity analysis did not change the results. Amongst the several factors that can predict OS, patient comorbidities and cancer stage have a high degree of precedence. Boakye et al (36) have shown that CRC patients with a greater number of comorbidities have significantly higher overall and cancer-specific mortality. Furthermore, advanced CRC i.e. stage IV cancer is already a well-known risk factor for poor OS (31). A subgroup analysis was performed based on adjustment of comorbidities and cancer stage by the included studies, however, the results still consistently demonstrated poor OS with frailty.

A major drawback in the pooled analysis was the differences in the frailty assessment scales used by the studies which could have led to variations in the number of patients identified as frail. Nevertheless, such inconsistencies have been noted in previous reviews on CRC (4, 36) and even for other malignancies (7, 8). Several measurements of frailty have been reported in literature which include and not limited to Identification of Seniors at Risk, the Groningen Frailty Indicator (GFI), Vulnerable Elders Survey-13, Triage Risk Screening Tool, the Clinical Frailty Scale (CFS), Fried criteria or the Rockwood index, the Geriatric 8 questionnaire, and modified frailty scale (37). Each of these tools uses different constructs to assess frailty. Like for example the CFS which is a pictorial-driven screening tool provides a global assessment of frailty and is easily used by physicians during routine examinations. The GFI is a 15-point self-assessment questionnaire that screens for limitations and classifies patients as frail and non-frail (37). The five-point modified frailty index combines four comorbidities (pulmonary disease, congestive heart failure, diabetes mellitus, and hypertension) and one functional status and is a reasonable frailty indicator (18). Research shows that the differences in the assessment tools alter the strength of the association between frailty and outcomes in different scenarios with some measurements demonstrating better predictive ability than others (5). In the current review, the included studies used 10 different measurement tools to assess frailty but despite the variations most of the individual studies noted a significant association between frailty and poor OS/DFS. Future research should be directed at identifying the best measurement tool for CRC patients. Also, there is a need for a unified frailty assessment scale for future research on CRC to improve comparability of outcomes across different populations and improve the evidence on the association between frailty and survival after cancer.

There could be several reasons why frailty could lead to poor long-term outcomes in CRC. Frailty has been identified as a major risk factor for postoperative surgical complications (4) and also for higher chemotherapy-related toxicity (6). Early short-term mortality and treatment-related toxicity could be important variables affecting long-term prognosis. Also, owing to the higher risk of early surgical complications, frail CRC patients may not receive complete surgical resection which may cause poor OS (38). Frail patients have increased levels of C-reactive protein and interleukin-6, indicating the role of chronic inflammation in its pathogenesis (39). Local immune response and baseline systemic inflammation have a major role in cancer progression and recurrence (40). Furthermore, malnutrition is an important contributor to frailty and poor nutritional status has been an important prognostic indicator in CRC (41, 42).

Despite frailty being an inevitable age-related condition with reduced function of multiple physiological systems, there have been attempts to improve frailty and subsequently the prognosis of CRC. Prehabilitation programs have been developed which consist of customized exercises under the supervision of experts or home-based aerobic and resistance training programs. Also, nutritional support is provided to frail patients to ascertain adequate protein and energy intake. Some programs also include correction of anemia, tobacco and alcohol cessation, and pharmacotherapy (43). Overall, research shows that prehabilitation in patients undergoing CRC surgery can reduce the risk of short-term complications and reduce the length of hospital stay (44). However, given the results of this review which demonstrate significant long-term implications of frailty on CRC outcomes; it is essential that further studies are undertaken to examine the effects of prehabilitation programs on long-term outcomes. Frail patients should be prioritized during cancer management and appropriately counselled regarding the risk of poor prognosis.

## Limitations

There are several limitations to this review. Importantly, as discussed earlier, the variation in frailty assessment tools is a major drawback that could have skewed the review results. It is still unclear which measurement tool best predicts the prognosis of CRC. Secondly, patients with several different cancer stages and undergoing different treatments were included. A subgroup analysis was possible only for studies including and excluding stage IV disease. The current review could not separately assess the prognostic value of frailty for stage I, II, and III disease. Thirdly, the observational nature of the data which was mainly derived from medical records also has an inherent bias that could not be negated. Fourthly, only five studies reported data on DFS and a subgroup analysis could not be conducted due to limited data. Fifthly, despite most studies using adjusted data, there were many variations in the confounders analyzed. Other unknown and known confounders that were not adjusted may have influenced the prognosis of CRC. Lastly, it was noted that all studies showed a positive association between frailty and poor outcomes after CRC. While the funnel plot showed no publication bias, the possibility of selective reporting of positive results and publication of only significant outcomes cannot be negated. The current review did not search gray literature and unpublished results were not included. It is plausible that non-significant results were not published and therefore not included in this review.

## Conclusions

Our results show that frail CRC patients have poor OS and DFS as compared to non-frail patients. Variations in frailty measurement tools and high inter-study heterogeneity are major limitations of the review. There is a need for a unified frailty assessment approach in future colorectal cancer research to reduce variability of studies. Also, future trials should focus on development and testing of modular prehabilitation programs to improve long-terms outcomes of CRC.

## Data availability statement

Publicly available datasets were analyzed in this study. This data can be found here: Databases of PubMed, Embase, and Web of Science were searched online for English-language articles.

## Author contributions

JH: Conceptualization, Data curation, Investigation, Software, Writing – original draft. QZ: Data curation, Investigation, Supervision, Validation, Writing – review & editing. JRL: Conceptualization, Formal Analysis, Methodology, Project administration, Resources, Writing – original draft. FY: Conceptualization, Data curation, Funding acquisition, Supervision, Writing – original draft. JL: Conceptualization, Data curation, Investigation, Methodology, Project administration, Writing – review & editing.

## Supplementary material

Supplementary Figure 1 Funnel plot to assess publication bias.

## References

[1] Pérez-Escalante E Cariño-Cortés R Fernández-Martínez E Ortiz MI Muñoz-Pérez VM Sánchez-Crisóstomo I . Colorectal cancer: causes and evidence of chemopreventive treatments. Curr Pharm Biotechnol (2018) 19:1135–55. doi: 10.2174/1389201020666181226112712 30585544

[2] Brenner H Chen C . The colorectal cancer epidemic: challenges and opportunities for primary, secondary and tertiary prevention. Br J Cancer (2018) 119:785–92. doi: 10.1038/S41416-018-0264-X PMC6189126 30287914

[3] Bray F Ferlay J Soerjomataram I Siegel RL Torre LA Jemal A . Global cancer statistics 2018: GLOBOCAN estimates of incidence and mortality worldwide for 36 cancers in 185 countries. CA Cancer J Clin (2018) 68:394–424. doi: 10.3322/CAAC.21492 30207593

[4] Cai M Gao Z Liao J Jiang Y He Y . Frailty affects prognosis in patients with colorectal cancer: A systematic review and meta-analysis. Front Oncol (2022) 12:1017183. doi: 10.3389/FONC.2022.1017183 36408138 PMC9669723

[5] Aguayo GA Vaillant MT Donneau AF Schritz A Stranges S Malisoux L . Comparative analysis of the association between 35 frailty scores and cardiovascular events, cancer, and total mortality in an elderly general population in England: An observational study. PloS Med (2018) 15:e1002543. doi: 10.1371/JOURNAL.PMED.1002543 29584726 PMC5870943

[6] Retornaz F Guillem O Rousseau F Morvan F Rinaldi Y Nahon S . Predicting chemotherapy toxicity and death in older adults with colon cancer: results of MOST study. Oncologist (2020) 25:e85–93. doi: 10.1634/THEONCOLOGIST.2019-0241 PMC6964155 31387952

[7] Wang S Yang T Qiang W Shen A Zhao Z Yang H . The prevalence of frailty among breast cancer patients: a systematic review and meta-analysis. Support Care Cancer (2022) 30:2993–3006. doi: 10.1007/S00520-021-06641-8 34694496

[8] Komici K Bencivenga L Navani N D’Agnano V Guerra G Bianco A . Frailty in patients with lung cancer: A systematic review and meta-analysis. Chest (2022) 162:485–97. doi: 10.1016/J.CHEST.2022.02.027 35217002

[9] Vitzthum LK Feng CH Noticewala S Hines PJ Nguyen C Zakeri K . Comparison of comorbidity and frailty indices in patients with head and neck cancer using an online tool. JCO Clin Cancer Inf (2018) 2:1–9. doi: 10.1200/CCI.18.00082 30652602

[10] Hersh AM Pennington Z Hung B Patel J Goldsborough E Schilling A . Comparison of frailty metrics and the Charlson Comorbidity Index for predicting adverse outcomes in patients undergoing surgery for spine metastases. J Neurosurg Spine (2021) 36:849–57. doi: 10.3171/2021.8.SPINE21559 34826820

[11] Page MJ McKenzie JE Bossuyt PM Boutron I Hoffmann TC Mulrow CD . The PRISMA 2020 statement: An updated guideline for reporting systematic reviews. Int J Surg (2021) 88:105906. doi: 10.1016/j.ijsu.2021.105906 33789826

[12] Wells G Shea B O’Connell D Peterson J Welch V Losos M . The Newcastle-Ottawa Scale (NOS) for assessing the quality of nonrandomised studies in meta-analyses . Available at: http://www.ohri.ca/programs/clinical_epidemiology/oxford.asp (Accessed October 30, 2020).

[13] Tierney JF Stewart LA Ghersi D Burdett S Sydes MR . Practical methods for incorporating summary time-to-event data into meta-analysis. Trials (2007) 8:16. doi: 10.1186/1745-6215-8-16 17555582 PMC1920534

[14] Neuman HB O’Connor ES Weiss J Loconte NK Greenblatt DY Greenberg CC . Surgical treatment of colon cancer in patients aged 80 years and older: analysis of 31,574 patients in the SEER-Medicare database. Cancer (2013) 119:639–47. doi: 10.1002/CNCR.27765 PMC3502632 22893570

[15] Aaldriks AA van der Geest LGM Giltay EJ le Cessie S Portielje JEA Tanis BC . Frailty and malnutrition predictive of mortality risk in older patients with advanced colorectal cancer receiving chemotherapy. J Geriatr Oncol (2013) 4:218–26. doi: 10.1016/J.JGO.2013.04.001 24070460

[16] Bensken WP Schiltz NK Warner DF Kim DH Wei MY Quiñones AR . Comparing the association between multiple chronic conditions, multimorbidity, frailty, and survival among older patients with cancer. J Geriatr Oncol (2022) 13:1244–52. doi: 10.1016/J.JGO.2022.06.011 PMC9798334 35786369

[17] Chen LJ Nguyen TNM Chang-Claude J Hoffmeister M Brenner H Schöttker B . Incorporation of functional status, frailty, comorbidities and comedication in prediction models for colorectal cancer survival. Int J Cancer (2022) 151:539–52. doi: 10.1002/IJC.34036 35435251

[18] Ogata T Sadakari Y Nakane H Koikawa K Kanno H Kohata R . The five-item modified frailty index predicts long-term outcomes in elderly patients undergoing colorectal cancer surgery. World J Surg Oncol (2023) 21:268. doi: 10.1186/S12957-023-03150-2 37626381 PMC10463643

[19] Abdelfatah E Ramos-Santillan V Cherkassky L Cianchetti K Mann G . High risk, high reward: frailty in colorectal cancer surgery is associated with worse postoperative outcomes but equivalent long-term oncologic outcomes. Ann Surg Oncol (2023) 30:2035–45. doi: 10.1245/S10434-022-12970-7 36648616

[20] Liposits G Ryg J Skuladottir H Winther SB Möller S Hofsli E . Prognostic value of baseline functional status measures and geriatric screening in vulnerable older patients with metastatic colorectal cancer receiving palliative chemotherapy - The randomized NORDIC9-study. J Geriatr Oncol (2023) 14:101408. doi: 10.1016/J.JGO.2022.11.007 36494261

[21] Ommundsen N Wyller TB Nesbakken A Jordhøy MS Bakka A Skovlund E . Frailty is an independent predictor of survival in older patients with colorectal cancer. Oncologist (2014) 19:1268–75. doi: 10.1634/THEONCOLOGIST.2014-0237 PMC4257747 25355846

[22] Ugolini G Pasini F Ghignone F Zattoni D Reggiani MLB Parlanti D . How to select elderly colorectal cancer patients for surgery: a pilot study in an Italian academic medical center. Cancer Biol Med (2015) 12:302–7. doi: 10.7497/J.ISSN.2095-3941.2015.0084 PMC4706530 26779367

[23] Jorgensen ML Young JM Dobbins TA Solomon MJ . A mortality risk prediction model for older adults with lymph node-positive colon cancer. Eur J Cancer Care (Engl) (2015) 24:179–88. doi: 10.1111/ECC.12288 25660420

[24] van der Vlies E Smits AB Los M van Hengel M Bos WJW Dijksman LM . Implementation of a preoperative multidisciplinary team approach for frail colorectal cancer patients: Influence on patient selection, prehabilitation and outcome. J Geriatr Oncol (2020) 11:1237–43. doi: 10.1016/J.JGO.2020.04.011 32359885

[25] Mima K Miyanari N Morito A Yumoto S Matsumoto T Kosumi K . Frailty is an independent risk factor for recurrence and mortality following curative resection of stage I-III colorectal cancer. Ann Gastroenterol Surg (2020) 4:405–12. doi: 10.1002/AGS3.12337 PMC7382441 32724884

[26] Cespedes Feliciano EM Hohensee C Rosko AE Anderson GL Paskett ED Zaslavsky O . Association of prediagnostic frailty, change in frailty status, and mortality after cancer diagnosis in the women’s health initiative. JAMA Netw Open (2020) 3:e2016747. doi: 10.1001/JAMANETWORKOPEN.2020.16747 32926116 PMC7490646

[27] Tokuda K Morine Y Miyazaki K Yamada S Saito Y Nishi M . Frailty can predict prognosis after hepatectomy in patients with colorectal liver metastasis. Anticancer Res (2021) 41:4637–44. doi: 10.21873/ANTICANRES.15277 34475092

[28] Artiles-Armas M Roque-Castellano C Fariña-Castro R Conde-Martel A Acosta-Mérida MA Marchena-Gómez J . Impact of frailty on 5-year survival in patients older than 70 years undergoing colorectal surgery for cancer. World J Surg Oncol (2021) 19:106. doi: 10.1186/S12957-021-02221-6 33838668 PMC8037830

[29] Mahar AL Compton C Halabi S Hess KR Weiser MR Groome PA . Personalizing prognosis in colorectal cancer: A systematic review of the quality and nature of clinical prognostic tools for survival outcomes. J Surg Oncol (2017) 116:969–82. doi: 10.1002/JSO.24774 PMC5760443 28767139

[30] Gunderson LL Sargent DJ Tepper JE Wolmark N O’Connell MJ Begovic M . Impact of T and N stage and treatment on survival and relapse in adjuvant rectal cancer: a pooled analysis. J Clin Oncol (2004) 22:1785–96. doi: 10.1200/JCO.2004.08.173 15067027

[31] Edge SB Compton CC . The American Joint Committee on Cancer: the 7th edition of the AJCC cancer staging manual and the future of TNM. Ann Surg Oncol (2010) 17:1471–4. doi: 10.1245/S10434-010-0985-4 20180029

[32] Haq AI Schneeweiss J Kalsi V Arya M . The Dukes staging system: a cornerstone in the clinical management of colorectal cancer. Lancet Oncol (2009) 10:1128. doi: 10.1016/S1470-2045(09)70157-3 19880067

[33] Chen S Ma T Cui W Li T Liu D Chen L . Frailty and long-term survival of patients with colorectal cancer: a meta-analysis. Aging Clin Exp Res (2022) 34:1485–94. doi: 10.1007/S40520-021-02072-X 35103954

[34] Tsai H-H Yu J-C Hsu H-M Chu C-H Hong Z-J Feng A-C . The impact of frailty on breast cancer outcomes: evidence from analysis of the Nationwide Inpatient Sample, 2005-2018. Am J Cancer Res (2022) 12:5589–98. PMC9827099 36628280

[35] Alamgeer M Ling RR Ueno R Sundararajan K Sundar R Pilcher D . Frailty and long-term survival among patients in Australian intensive care units with metastatic cancer (FRAIL-CANCER study): a retrospective registry-based cohort study. Lancet Heal Longev (2023) 4:e675–84. doi: 10.1016/S2666-7568(23)00209-X 38042160

[36] Boakye D Rillmann B Walter V Jansen L Hoffmeister M Brenner H . Impact of comorbidity and frailty on prognosis in colorectal cancer patients: A systematic review and meta-analysis. Cancer Treat Rev (2018) 64:30–9. doi: 10.1016/J.CTRV.2018.02.003 29459248

[37] Goede V . Frailty and cancer: current perspectives on assessment and monitoring. Clin Interv Aging (2023) 18:505–12. doi: 10.2147/CIA.S365494 PMC10066705 37013130

[38] González-Senac NM Mayordomo-Cava J Macías-Valle A Aldama-Marín P González SM Arnés MLC . Colorectal cancer in elderly patients with surgical indication: state of the art, current management, role of frailty and benefits of a geriatric liaison. Int J Environ Res Public Health (2021) 18:6072. doi: 10.3390/IJERPH18116072 34199923 PMC8200127

[39] Zhang X Meng X Chen Y Leng SX Zhang H . The biology of aging and cancer: frailty, inflammation, and immunity. Cancer J (2017) 23:201–5. doi: 10.1097/PPO.0000000000000270 28731941

[40] Diakos CI Charles KA McMillan DC Clarke SJ . Cancer-related inflammation and treatment effectiveness. Lancet Oncol (2014) 15:e493–503. doi: 10.1016/S1470-2045(14)70263-3 25281468

[41] Noh GT Han J Cho MS Hur H Min BS Lee KY . Impact of the prognostic nutritional index on the recovery and long-term oncologic outcome of patients with colorectal cancer. J Cancer Res Clin Oncol (2017) 143:1235–42. doi: 10.1007/S00432-017-2366-X PMC11819198 28243747

[42] Valentini A Federici M Cianfarani MA Tarantino U Bertoli A . Frailty and nutritional status in older people: the Mini Nutritional Assessment as a screening tool for the identification of frail subjects. Clin Interv Aging (2018) 13:1237–44. doi: 10.2147/CIA.S164174 PMC6047619 30034227

[43] Tautenhahn HM Krautscheid A Schulte K Settmacher U Zanow J . [Prerehabilitation in frail patients: Frailty as a risk factor]. Chirurg (2020) 91:103–8. doi: 10.1007/S00104-019-01081-X 31828385

[44] Chang MC Choo YJ Kim S . Effect of prehabilitation on patients with frailty undergoing colorectal cancer surgery: a systematic review and meta-analysis. Ann Surg Treat Res (2023) 104:313. doi: 10.4174/ASTR.2023.104.6.313 37337603 PMC10277181

