# Supplementary figures and images for: Frailty worsens long-term survival in patients with colorectal cancer: a systematic review and meta-analysis
Source: Front Oncol. 2024 Feb 9;14:1326292. doi: 10.3389/fonc.2024.1326292 (PMC10889110; doi:10.3389/fonc.2024.1326292)

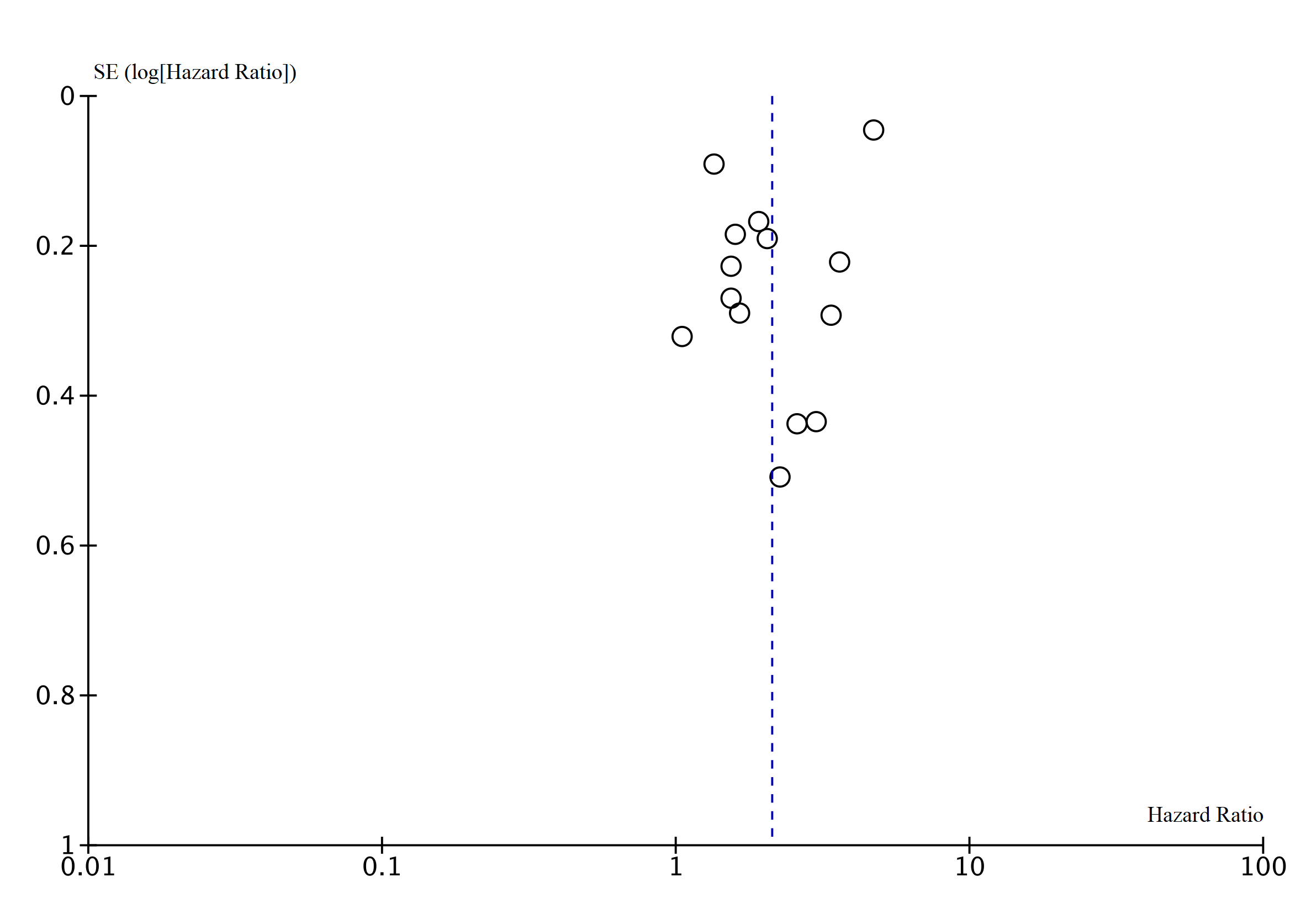

Supplement: Supplementary Figure 1 — Funnel plot to assess publication bias. [file Image_1.tiff]
